# The Relationship Between Anxiety and Employment Status in a German Working-Age Population: Findings on Sex-Specific Prevalence Rates and Associated Factors of Anxiety From the LIFE-Adult-Study

**DOI:** 10.1155/da/1883623

**Published:** 2025-07-30

**Authors:** Charlyn Görres, Alexander Pabst, Andrea E. Zülke, Heide Glaesmer, Andreas Hinz, Christoph Engel, Toralf Kirsten, Nigar Reyes, Markus Loeffler, Steffi G. Riedel-Heller, Margrit Löbner

**Affiliations:** ^1^Institute of Social Medicine, Occupational Health and Public Health (ISAP), Medical Faculty, University of Leipzig, Leipzig, Germany; ^2^Department of Medical Psychology and Medical Sociology, Medical Faculty, University of Leipzig, Leipzig, Germany; ^3^Institute of Medical Informatics, Statistics and Epidemiology, University of Leipzig, Leipzig, Germany; ^4^LIFE - Leipzig Research Centre for Civilization Diseases, University of Leipzig, Leipzig, Germany; ^5^Department for Medical Data Science, University Hospital Leipzig, Leipzig, Germany

**Keywords:** anxiety, GAD-7, mental health, population-based, prevalence, sex differences, unemployment

## Abstract

**Introduction:** Anxiety disorders are among the most prevalent mental illnesses worldwide, with a 12-month prevalence rate of 14%–15.3%. Prior research has demonstrated a correlation between unemployment and impaired mental health. The primary objective of this study is to examine the relationship between anxiety and employment status in Germany.

**Methods:** The sample consisted of 4885 working-age participants (18–65 years) from the baseline survey of a population-based cohort study. Anxiety was assessed using the General Anxiety Disorder Scale-7 (GAD-7). Employment status was divided into four groups: full-time employment (FTE group), part-time employment (PTE group), ALG I (ALG I group, being unemployed receiving entitlement-based benefits), and ALG II (ALG II group, being unemployed receiving means-tested benefits). Negative binomial regressions were conducted to examine the association between anxiety, employment status, sociodemographic variables (age, sex, marital status, and education), and social resources (Lubben Social Network Scale 6 [LSNS-6]).

**Results:** The prevalence of anxiety symptoms (FTE group: 5.0%, PTE group: 4.8%, ALG I group: 2.7%, and ALG II group: 17.6%) differed between employment status groups. The ALG II group exhibited a markedly elevated prevalence compared to all other groups (*p*  < 0.001). The prevalence of anxiety symptoms was higher among females in the FTE group (7.6%) compared to males (2.9%) in the same category (*p*  < 0.001). The initial regression model indicated a statistically significant association between anxiety symptoms and the ALG II group (*p*  < 0.001) and the PTE group (*p*=0.006). After including additional variables, this effect maintained its statistical significance. Factors associated with a higher risk of anxiety symptoms were female sex (*p*  < 0.001), low educational level (*p*=0.007), and less social resources (*p*  < 0.001).

**Conclusion:** Unemployed individuals who receive ALG II are at the highest risk of developing anxiety symptoms among those who are able to work. These findings suggest the need for targeted psychosocial and occupational support for unemployed individuals receiving ALG II.

## 1. Introduction

Approximately 61.5 million individuals in Europe suffer from anxiety disorders annually [1]. Anxiety disorders are the most prevalent mental illnesses, with a 12-month prevalence of approximately 15.3% in Germany [2, 3].

Previous research has shown that unemployment is associated with poor mental health, which is supported by the findings of Gündel et al. [4], Maurer et al. [5], Paul and Zechmann [6], and Hollederer [7]. A meta-analysis by Paul and Moser [8] found that the unemployed are twice as likely to suffer from mental illness compared to those who are employed (34% vs. 16%). Other studies have found that unemployed individuals experience higher levels of anxiety symptoms compared to those who are employed [9–11].

The mechanisms underlying this observation have been studied extensively. Research suggests that unemployment can both result from and contribute to psychological distress [10, 12]. For instance, Jahoda's latent deprivation model [10, 13] attempts to explain the adverse effects of unemployment on mental health. According to Jahoda's model, employment serves five latent functions: providing structure to one's time, facilitating social interaction, fostering a sense of collective purpose, establishing social identity/status, and promoting activity. Unemployment results in the loss of employment functions, which can have negative impacts on mental health. Therefore, employment plays a crucial role in satisfying human needs and maintaining health [14–16].

Considerations regarding the increased risk of losing one's job or remaining unemployed for those experiencing psychological stress are based on the selection theory [10, 12]. Additionally, prolonged unemployment can increase the risk of suicide, anxiety, and depression [17, 18]. In Germany, where the current unemployment rate is 5.5%, a significant proportion of individuals is unemployed [19].

Previous studies on unemployment and anxiety have not distinguished between two groups of unemployment in Germany: entitlement-based benefits, in German “Arbeitslosengeld I” (ALG I), and means-tested benefits, in German “Arbeitslosengeld II” (ALG II) since 2023 “Bürgergeld.” These two groups differ in terms of the length and extent of financial support, as well as obligations to the job center. For instance, individuals who have worked for at least 1 year in the last 2 years are eligible for unemployment insurance benefits for 1 year and, therefore, belong to the group of unemployed individuals receiving entitlement-based benefits (ALG I). All other persons who have worked less in the last 2 years or whose unemployment insurance benefits are ending can receive means-tested benefits (ALG II). To be eligible for ALG II, recipients must be available to the job center at all times and willing to accept any suitable job offer [20].

Zülke et al. [21] demonstrated an increased risk of depression symptoms in the group of unemployed receiving ALG II. The authors noted that the group of unemployed people is not homogeneous and requires further investigation to develop appropriate prevention and treatment approaches. This sentiment is echoed by Arena et al. [18], who emphasize the need for interventions and further research into anxiety symptoms among the unemployed. This need is supported by the rising rates of anxiety disorders over time [22] and a greater stigmatization of unemployed people who receive ALG II/means-tested benefits compared to those who receive ALG I/entitlement-based benefits, since the latter are based upon previous contributions and express an “earned” right [21]. Additionally, it is important to consider higher replacement rates of unemployment benefits as a buffer against the negative health effects of unemployment. This is especially true for those receiving ALG I compared to those receiving ALG II, as noted by Voßemer et al. [23] and Zülke et al. [21].

No other sociodemographic factors, such as sex, age, education, and social support, have been examined in the association between anxiety and (un)employment. However, other studies suggest the importance of these variables in the association between unemployment and anxiety symptoms [6, 24, 25]. For instance, there is no clear evidence of the influence of sex. Some studies have found that unemployed females experience higher levels of anxiety compared to unemployed males [25, 26]. However, Paul and Zechmann [6] have cited studies that contradict this finding or suggest that further research is needed. To date, no study has investigated the possible association between different groups of unemployment and anxiety symptoms, despite anxiety symptoms being one of the most frequent psychological impairments in Germany.

The primary aim of this study is to examine the relationship between anxiety symptoms and employment status among individuals of working age (18–65 years) in Germany. This relationship will be analyzed in conjunction with socioeconomic and sociodemographic factors in a population-based sample.

This study aims to answer the following research questions:

1. What is the prevalence of anxiety among the following four employment status groups: (a) unemployed individuals receiving ALG I, (b) unemployed individuals receiving ALG II, (c) part-time employed individuals, and (d) full-time employed individuals?
2. Is there a difference in the prevalence of anxiety among these groups, based on sex?
3. How are anxiety symptoms associated with employment status (ALG I, ALG II, part-time employment [PTE], full-time employment [FTE]), as well as other sociodemographic variables and social resources?

## 2. Material and Methods

### 2.1. Sample

**Figure 1:**
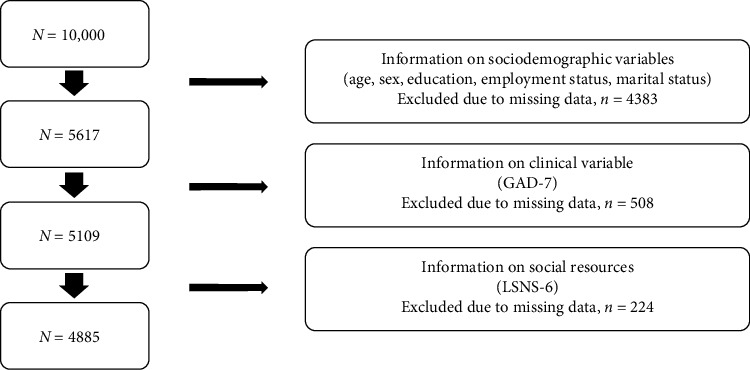
Flowchart of the study sample (baseline assessment: 2011–2014). *N*/*n* = sample size. GAD-7, General Anxiety Disorder Scale-7; LSNS-6, short version of the Lubben Social Network Scale-6.

The sample was obtained from a population-based large-scale cohort study (LIFE-Adult-Study) in Germany, using the baseline survey (*N* = 10,000; assessment period: 2011–2014) [27, 28]. The study included participants aged 18–79 years. For more details on the study design, see Loeffler [27] and Engel et al. [28]. The study sample flow chart for our analysis is depicted in Figure 1. Only data from people of working age (18–65 years) and with full information on employment status and relevant sociodemographic variables (education and marital status) were included (*N* = 5617). In the next step, *n* = 508 respondents were excluded due to missing values on the General Anxiety Disorder Scale-7 (GAD-7). In the final step, *n* = 224 respondents were excluded due to missing values in the Lubben Social Network Scale 6 (LSNS-6). This resulted in a final sample size of *N* = 4885.

Sensitivity analyses revealed that the proportion of missing values for the individual variables ranged from 0% to 6.6% in the final study sample. The missing values analysis revealed that older respondents (*p*  < 0.001) were significantly more likely to have missing values. In contrast, respondents with higher levels of education were significantly less likely to have missing values (*p*=0.023).

### 2.2. Measures

#### 2.2.1. Anxiety Symptoms

Anxiety symptoms were measured using the German version [9] of the GAD-7 [29]. The GAD-7 consists of seven items, representing a unidimensional scale [10]. The questionnaire measures the following core symptoms experienced in the last 2 weeks: restlessness, tension, irritability, and rumination. The seven items are answered using a four-point Likert scale ranging from 0 = “not at all” to 3 = “almost every day.” The sum score is then calculated, with a higher score (maximum 21) indicating a higher level of anxiety symptoms related to GAD symptoms. Spitzer et al. [29] propose a cutoff value for GAD symptoms of ≥10, which achieves a best possible sensitivity (89%) and specificity (82%).

#### 2.2.2. Employment Status

Participants answered questions about their employment status using standardized questionnaires. The question asked was, “Are you currently employed?” Employment includes all paid activities, regardless of duration. Four employment status groups were formed to distinguish between types of employment: FTE or PTE, unemployment with ALG I, and unemployment with ALG II. Full-time was defined as working 35 h or more per week. Part-time was defined as working 15–34 h per week. Individuals, who have worked for at least 1 year in the last 2 years are eligible for unemployment insurance benefits for 1 year and, therefore, belong to the group of unemployed individuals receiving entitlement-based benefits (ALG I). All other persons who have worked less in the last 2 years or whose unemployment insurance benefits are ending can receive means-tested benefits (ALG II). In accordance with Bartoll et al. [30], we now use the following abbreviations: full-time employment group = FTE group, part-time employment group = PTE group, unemployment with ALG I = ALG I group, and unemployment with ALG II = ALG II group.

#### 2.2.3. Other Measures

To consider the multifactorial relationship between employment status and health, sociodemographic variables such as sex assigned at birth (female and male), age (continuous), marital status (married and living together, married and living separately, single, divorced, or widowed), and education according to Comparative Analysis of Social Mobility in Industrial Nations (CASMIN; low, middle, and high) were included [6, 8]. The CASMIN scale [31] was used to collect information on general and occupational qualifications.

Additionally, the study included an analysis of the potential association of low social resources, using the short version of the LSNS-6 [32]. This questionnaire measures perceived social support, availability of social contacts, and social initiative. Participants are asked to indicate the number of relatives and friends they feel close to and could call on for help, as well as the number of relatives and friends they see or hear from at least once a month. Responses are recorded on a five-point Likert scale ranging from 0 (none) to 5 (nine or more) for each of the six items. The sum score ranges from 0 to 30. Increasing scores indicate higher levels of social resources. According to Lubben et al. [32], a score of less than 12 points indicates social isolation.

### 2.3. Statistical Analyses

Descriptive and inferential statistical analyses were conducted using Stata (SE) 17 and SPSS 29.0 software. To examine possible differences between employment status groups, one-way analysis of variance (ANOVA) and Chi^2^-tests were used. As the dependent variable (sum score of GAD-7) did not follow a normal distribution, negative binomial regressions were calculated to address the third research question. To test the association between employment status and anxiety symptoms, three models were compared hierarchically: the unadjusted model (Model 1) was compared with the sociodemographic variables model (Model 2), which additionally included education, age, sex, and marital status; the social variable model (Model 3) additionally included social resources (LSNS-6). Logistic regressions were conducted to examine differences in anxiety prevalence by sex. The tables of results can be found in the Supporting Information (Tables S1 and S2). In addition, an additional negative binomial regression (Table S3) was conducted, including depressive symptoms as an additional predictor variable, to examine the potential association between depressive and anxiety symptoms, taking into account the high comorbidity between the two [33–35] (Table S3). First, the entire sample was analyzed and then, employment status groups were compared, including the FTE group, the PTE group, the ALG I group, and the ALG II group. Additionally, Wald tests were performed to assess the overall significance of categorical variables with more than two categories such as employment status, education, and marital status. For all analyses, a significance level of *p*  < 0.05 (two-tailed) was used. For post hoc tests (prevalence comparisons), a Bonferroni adjustment was made resulting in a significance level of *p*  < 0.008. Either odds ratios (ORs) or incidence-rate ratios (IRRs) were used to present risks in the analyses, as appropriate. The analyses were based on a 95% confidence interval (CI). The sample was weighted by age and sex using German census data from 2011. In the following tables, all figures are weighted except for the frequencies.

## 3. Results

### 3.1. Sample Characteristics

Table 1 shows the sample characteristics of the study for the total sample and by employment status group. All employment status groups, except for the comparison between the ALG II group and the ALG I group, differed significantly from each other in terms of the average age (*p*  < 0.001). The ALG I group had the highest average age at *M* = 48.2 years, SD = 12.6. On the other hand, those in the PTE group had the lowest average age at *M* = 42.8 years, SD = 10.7. Additionally, there are significant differences in the sex distribution between the PTE group and all other employment status groups (*p*  < 0.001), with the highest proportion of females (79.9%) being in the employed part-time group. There were significant differences in marital status between the ALG II group and all other employment status groups (*p*  < 0.001). The ALG II group had the largest proportion of singles (44.1%), divorced (30.3%), and separated living married (3.8%) and the smallest proportion in the other marital statuses (widowed (1.8%) and married living together (3.8%)). The variable for education indicated that the ALG II group differed significantly from all other employment status groups (*p*  < 0.001). This group has the highest proportion of a low educational level (13.4%) and middle educational level (67.5%) participants and the smallest proportion of participants with a high education level (19.1%). The analysis of the LSNS-6 revealed that for the ALG II group social resources (*M* = 13.3, SD = 5.4) were significantly lower compared to all other employment status groups (*p*  < 0.001).

### 3.2. Prevalence of Anxiety

Table 2 shows that the prevalence of anxiety symptoms, defined by a GAD-7 sum score ≥10, was 5.6% for the overall sample. The prevalence was 5.0% for the FTE group, 4.8% for the PTE group, 2.7% for the ALG I group, and 17.6% for the ALG II group. The prevalence rate for the ALG II group was almost four times higher than for all other employment status groups. Post hoc tests showed that these differences were significant (*p*  < 0.001).

### 3.3. Sex-Specific Prevalence Rates of Anxiety Symptoms According to Employment Status

Table 2 shows that from the overall sample the prevalence of anxiety symptoms was 3.6% for males and 7.6% for females. Females in the ALG II group had by far the highest prevalence rate of anxiety symptoms at 22.0%. The lowest prevalence was among males in the ALG I group at 1.9%.

The association of sex with the prevalence of anxiety was revealed through binary logistic regressions (Table S1). The results indicate that female participants had a significantly higher risk of experiencing anxiety symptoms compared to male participants in the overall sample (*p*  < 0.001). This association was also observed in the FTE group (*p*  < 0.001), but not in the other employment status groups.

### 3.4. Factors Associated With Anxiety Symptoms in the Working-Age Sample

As shown in Table 3, the first negative binomial regression model indicated a significantly higher risk of anxiety symptoms for the PTE group (*p*=0.006) and the ALG II group (*p*  < 0.001) compared to the FTE group. Employment status was also globally significant (*p*  < 0.001). After adding relevant sociodemographic variables (Model 2), an increased risk of anxiety symptoms remained only for the ALG II group (*p*  < 0.001). Employment status remained globally significant (*p*  < 0.001). Additionally, the analysis revealed that female participants had a significantly higher risk of anxiety symptoms compared to male participants (*p*  < 0.001). In contrast, the results showed that participants with a high educational level (*p*  < 0.001) and middle educational level (*p*  < 0.001) had a significantly lower risk of anxiety symptoms compared to those with low educational level. Education was globally significant (*p*  < 0.001). The model also showed a significantly higher risk of anxiety symptoms for participants who were married and living separately (*p*=0.022) or divorced (*p*=0.008) compared to those who were married and living together. Marital status was also globally significant (*p*=0.016). In Model 3, which incorporates an additional social variable, the relationship between employment status and anxiety symptoms remained statistically significant. Model 3 also demonstrated a higher risk of anxiety symptoms in female participants compared to male participants (*p*  < 0.001). Education (*p*=0.007) became a significant predictor as well. Moreover, higher levels of social resources were associated with a lower risk of anxiety symptoms (*p*  < 0.001).

We further investigated potential differences regarding the association of unemployment and anxiety symptoms between males and females by conducting stratified negative binomial regression analyses (Model 3) by sex. The table of results can be found in the Supporting Information (Table S2). In the ALG II group, a significantly higher risk of anxiety symptoms was observed in both sexes when compared to individuals in the FTE group (females *p*=0.015 and males *p*=0.001). Moreover, the results indicate that males in the PTE group exhibit an elevated risk of anxiety symptoms compared to males in the FTE group (*p*=0.013). Employment status held global significance only for males (*p* < 0.001), but not for females (*p*=0.060). Sex differences were also evident at higher levels of education. Specifically, males with higher educational level exhibited a significantly lower risk of anxiety symptoms compared to males with lower educational level (*p*=0.036). This was not the case for females (*p*=0.055).

To account for a possible association between depression and anxiety [33–35], an additional negative binomial regression (Table S3) was conducted, including depressive symptoms as an additional predictor variable. When accounting for depression, there was a significant increase in the risk of anxiety symptoms with higher depression sum scores (*p*  < 0.001). Concurrently, the employment status lost its significance (*p*=0.167). Further details are provided in the Supporting Information.

## 4. Discussion

### 4.1. Employment Status-Related Differences in Anxiety Prevalence Rates

This study examined the association between employment status and anxiety symptoms in a large population-based sample from Germany. The results showed that the prevalence of anxiety symptoms was significantly higher among the ALG II group (17.6%) than among the ALG I group (2.7%), the FTE group (5.0%), and the PTE group (4.8%). Females in the FTE group had a significantly higher risk of anxiety symptoms than males in the FTE group. Differences in prevalence of anxiety symptoms between employment status groups were partially explained by other variables, for example, sex.

This study represents the first empirical evidence on the prevalence of anxiety symptoms among the FTE group, the PTE group, the ALG I group, and the ALG II group in the German working-age population (18–65 years). Given the distinctive German unemployment benefit system, the significance of the current study is readily apparent. This allows in-depth insights on the prevalence of anxiety symptoms across different employment status categories.

In particular, a significantly higher prevalence of anxiety symptoms was found for unemployed people receiving ALG II. Similar findings have been observed in other studies conducted in Germany, which have demonstrated a higher prevalence of anxiety symptoms among unemployed individuals (receiving ALG I and II) compared to those in employment (full-time and part-time) [8, 36, 37].

Due to the specific conditions of the German unemployment benefit system, it is challenging to make direct comparisons with studies from other countries. Nevertheless, other studies from other countries corroborate the results found here, indicating that unemployment is also associated with higher anxiety prevalence and more anxiety symptoms [25, 38–43].

### 4.2. The Role of Full-Time Work and Female Sex in Relation to Anxiety Symptoms

The observation that employed females are more likely to experience anxiety symptoms than employed males is consistent with the empirical evidence indicating that females are more likely to experience anxiety symptoms than males in general. This relationship has been repeatedly demonstrated in research studies [2, 44–47]. One explanation for why the association was only found for the FTE group could be the distribution of the sample. The largest part of the sample was the FTE group. It is similarly plausible that inequalities in employment for females are more pronounced than for males, particularly among those who work full-time. The following are examples of such discriminatory practices: (1) lower pay, the so-called “gender pay gap,” (2) unequal distribution across different occupations (horizontal segregation), and (3) poorer opportunities for promotion within the company (vertical segregation) for females [48, 49]. The gender care gap, in which females in Germany work more than half as long as males in the care sector (in addition to their paid work), also represents a double burden for females [50]. It can be reasonably deduced that the aforementioned inequalities will indirectly impact mental health, in this case manifesting as anxiety symptoms.

Furthermore, a study by Berth et al. [51] revealed a markedly higher prevalence of perceived job insecurity among females (30%) in comparison to males (13%). Additionally, Zok [52] found that the fear of making mistakes at work is significantly more prevalent among employed females (37%) than among employed men (30.3%). This study also revealed that the fear of being bullied in the workplace was significantly more prevalent among female employed individuals (24.2%) than among male employed individuals (15.5%). The findings presented here, in conjunction with the higher prevalence rates of anxiety observed among employed females, indicate that employed females may experience greater stress at work than employed males. This stress can manifest in a number of ways, including anxiety symptoms.

In addition to the work context, numerous studies and investigations have been conducted in the research literature on gender and sex differences in anxiety disorders [53–55]. For example, in their review, Farhane-Medina et al. [54] identified factors that attempt to explain gender and sex differences in the prevalence of anxiety. For instance, several biological factors have been discussed in relation to sex differences in the expression of anxiety symptoms. These include the influence of the menstrual cycle on anxiety [56], sex-specific activation of brain structures in fear conditioning [57], and a gene-specific risk for female individuals [58].

As previously mentioned, psychosocial factors have been linked to the higher prevalence of anxiety symptoms in females in other studies [59, 60]. These studies have considered the influence of gender socialization and associated gender roles.

When interpreting gender and sex effects, however, a possible bias in the results due to the male tendency for self-reliance over help-seeking when dealing with anxiety problems should also be considered, with the possible consequence of underestimated anxiety prevalence in males [61]. The presence of gender-specific symptoms is also a consideration in the diagnosis of anxiety disorders, and thus, in their prevalence [62, 63].

In conclusion, it can be stated that the relationship between sex, gender, and health is a complex process that requires further investigation in order to gain a deeper understanding and develop effective prevention and treatment strategies.

### 4.3. The Link Between Education and Marital Status and Anxiety Symptoms

If the focus is now shifted away from a dichotomous view of anxiety (prevalence) towards a continuum (no anxiety symptoms to many anxiety symptoms), the results (Model 1) initially demonstrated a similarity to those of the prevalence of anxiety.

Employment status was found to be a significant factor related to the expression of anxiety symptoms. Upon closer examination, it became evident that the PTE group and the ALG II group exhibited a markedly elevated risk of anxiety symptoms in comparison to the FTE group. The relationship between employment status and anxiety symptoms underwent a notable change with the incorporation of sociodemographic (Model 2) and social resources (Model 3). The significant association observed in the Models 2 and 3, indicating a higher risk of anxiety symptoms among females, is also consistent with the results and explanatory approaches previously listed.

Previously unconsidered in the analyses, Model 2 initially revealed that education was an associated factor for the experience of anxiety symptoms. It can be concluded that individuals with both a middle and a high level of education exhibited a lower risk of anxiety symptoms compared to those with a low level of education. These findings are consistent with numerous other studies that have identified education and the associated socioeconomic resources as a protective factor against mental illness [25, 43, 64, 65]. It is noteworthy that the education variable retained its significance even after including the variable for social resources (LSNS-6). These findings imply that education contributes to the expression of anxiety symptoms, in addition to the notable impact of social resources. The protective effect of higher educational level does appear to be applicable in this instance.

Another significant sociodemographic factor associated with the expression of anxiety symptoms was marital status (Model 2). Divorced and married people living separately had a higher risk of developing anxiety symptoms compared to married people living together (Model 2). This finding is also consistent with many similar results from previous research studies [43, 66, 67]. A key underlying mechanism that has been cited in numerous research studies is the greater social integration and support that married individuals tend to experience in comparison to unmarried individuals. After the LSNS-6 variable was included (Model 3, Table S2 in the Supporting Information), the association gained particular relevance for married males living separately. This subgroup may thus be considered a potential risk group for anxiety symptoms.

### 4.4. The Relationship Between Social Integration and Anxiety Symptoms

Another significant finding of the study was the strong association between the level of social integration (LSNS-6) and the severity of anxiety symptoms (Model 3). This association was demonstrated to be independent of sex, with higher levels of social resources being linked to a lower risk of anxiety. The present finding is consistent with previous research, suggesting that social isolation and anxiety are interconnected through a complex and multifaceted relationship [68]. On the one hand, limited social interaction may increase the risk of developing anxiety symptoms by elevating stress levels and impairing emotional regulation. On the other hand, individuals experiencing anxiety symptoms often face difficulties in maintaining interpersonal relationships, which may lead to a gradual disengagement from social activities [68]. Consequently, social integration acts as a key protective factor in managing anxiety symptoms, with social relationships offering a buffering effect when characterized by open communication and mutual support [69, 70].

The increased risk of anxiety symptoms observed among individuals receiving ALG II suggests reduced social integration across various domains, potentially leading to a lack of protection against anxiety. Further research is needed to investigate this association more comprehensively.

### 4.5. The Relationship Between ALG II and Anxiety Symptoms

The present study shows that, in addition to the association between sociodemographic and social variables, employment status is significantly linked to anxiety symptoms. Specifically, individuals receiving ALG II exhibit an elevated risk of experiencing anxiety symptoms. In this context, previous research suggests that existential fears, concerns about the future, and stigmatization may significantly contribute to the development of anxiety symptoms [71].

The latter is widely regarded as a key factor affecting the mental health of individuals experiencing unemployment [72, 73]. It has been shown that the more people with unemployment feel stigmatized, the worse their state of health [74]. Although the vast majority of people with unemployment have experienced stigmatization [75], there is a lack of research on social unemployment stigma [73]. In addition to social devaluation and structural discrimination, the phenomenon of self-stigmatization is also of significance [76]. This is characterized by the internalization of existing stereotypes about individuals experiencing unemployment. In order to counteract the stigmatization of individuals experiencing unemployment, antistigma initiatives are necessary that address the material, historical, and political dimensions of stigmatization [77].

### 4.6. The Role of Part-Time Work and Male Sex in Relation to Anxiety Symptoms

The results of the study demonstrated that employment status is not a universal risk or protective factor of anxiety symptoms. The analysis revealed that only males in the PTE group exhibited an elevated risk of anxiety symptoms compared to males in the FTE group. In comparison to FTE, part-time positions tend to offer a lower remuneration and fewer advancement prospects [78]. Such concerns can give rise to financial anxieties, with individuals potentially experiencing difficulties in, for example, providing adequately for their families. Additionally, the concern of losing prestige and career opportunities or being perceived as less ambitious may also influence part-time employed males [79, 80].

It would be beneficial to conduct further research to ascertain whether males who work part-time may be at an increased risk of developing anxiety symptoms.

## 5. Strengths and Limitations

One of the strengths of this study is the comprehensive population-based sample used to examine the research questions. Using a weighting factor, the data from the LIFE-Adult-Study provide results that are representative of the German adult population. The detailed recording of employment status (differentiation between ALG I and ALG II) is also one of the strengths of the study. Despite these strengths, there are also some limitations. The research questions were examined in a cross-sectional design. Therefore, no causal statements can be made about the relationship between employment and anxiety. Previous studies have indicated that poor health among the unemployed may be both a cause and a consequence of unemployment [8, 81].

A further limitation of the study is the lack of information on the duration of unemployment. Previous studies have already demonstrated significant associations between unemployment duration and mental health [8, 82].

In addition, the instruments used are self-reports. Although the GAD-7, a validated and widely used instrument, was used to assess anxiety symptoms, it is based on self-report by the study participants and may, therefore, be susceptible to bias. In addition, a missing values analysis revealed that various sociodemographic and socioeconomic variables, such as older age and lower educational level, were more likely to have missing values in the baseline sample, which may have biased the results. It is also important to consider the relatively small ALG I group when attempting to generalize the results. The variance explained by the included factors was low. Therefore, in addition to the mental health variables considered here, further physical and psychological factors should be investigated in order to ascertain the risk of both anxiety symptoms and unemployment.

### 5.1. Practical Implications

Recent studies show that interventions to improve the mental health of unemployed individuals are effective [83, 84]. An example of this is the pilot project “JOBS Program Germany,” in which various interventions are carried out over several weeks as part of a mentor training course [85]. The intervention consists of the following elements: job-search skills training, active teaching and learning methods, trained trainers for program delivery, supportive learning environment, and preparation for setbacks [86]. The program has been shown to have a positive impact on health, including a reduction in psychological stress related to unemployment, improved life satisfaction and reduced depressive symptoms [87].

Another example of health promotion for unemployed individuals is the ongoing Leipzig-Individual Placement and Support for People with Mental Illness (LIPSY) project [88].

In this project, long-term unemployed people with mental health problems receive low-threshold screening, psychological diagnostics, and comprehensive psychometrics with subsequent referral to the care system.

In summary, there are some initial interventions for the unemployed in Germany with a focus on mental health promotion. However, these interventions are only available for a limited period of time and are not implemented across the board. Furthermore, according to the findings of this study, sociodemographic characteristics need to be taken into account when developing interventions with regard to anxiety symptoms and, in particular, comorbidities.

## 6. Conclusion

The study revealed that anxiety symptoms were more prevalent in females, as well as in individuals with a low level of education and in individuals who had comorbid depressive symptoms. With respect to employment status, the cohort of part-time employed males demonstrated a heightened susceptibility to anxiety symptoms. Additionally, the prevalence rates indicated an elevated risk of anxiety symptoms among ALG II recipients. To gain further insight into the underlying causes and effects of anxiety symptoms and their association with sociodemographic, economic, and clinical variables, longitudinal studies are required. For the workplace, there should be a greater focus on providing mental health support for males in part-time positions, in order to more effectively address their concerns and fears, and adapt structures as needed to enhance mental well-being. It is also important to consider the group of ALG II recipients in future studies, as there may be an increased need for psychosocial and professional support in this population [89].

## Figures and Tables

**Table 1:** Sample characteristics—overall and by employment status group.

Variable	Overall, *n* = 4885	FTE group, *n* = 3664	PTE group, *n* = 769	ALG I group, *n* = 134	ALG II group, *n* = 318	Chi^2^/*F⁣*^*∗*^	*p*-Value
Sociodemographic variables							
Age in years, *M* (SD)	44.2 (11.04)	44.1 (10.9)	42.8 (10.7)	48.2 (12.6)	47.7 (11.4)	17.8	**<0.001**
Sex						319.0	**<0.001**
Male, *n* (%)	2258 (49.8)	1918 (56.1)	120 (20.1)	57 (48.0)	163 (53.3)		
Female, *n* (%)	2627 (50.2)	1746 (43.9)	649 (79.9)	77 (52.0)	155 (46.7)		
Education						101.1	**<0.001**
Low, *n* (%)	163 (4.0)	102 (5.3)	20 (2.8)	9 (7.6)	32 (13.4)		
Middle, *n* (%)	3002 (59.4)	2190 (61.5)	497 (54.7)	81 (57.3)	234 (67.5)		
High, *n* (%)	1720 (36.6)	1372 (33.2)	252 (42.5)	44 (35.1)	52 (19.1)		
Marital status						127.2	**<0.001**
Married (living together), *n* (%)	2611 (42.8)	2012 (43.9)	457 (46.1)	68 (39.7)	74 (20.0)		
Married (living separately), *n* (%)	158 (2.4)	115 (2.3)	22 (2.5)	5 (2.4)	16 (3.8)		
Single, *n* (%)	1224 (41.8)	919 (41.8)	166 (41.3)	28 (39.1)	111 (44.1)		
Divorced, *n* (%)	798 (11.9)	558 (11.0)	104 (8.7)	27 (15.8)	109 (30.3)		
Widowed, *n* (%)	94 (1.2)	60 (1.0)	20 (1.4)	6 (3.1)	8 (1.8)		
Social resources							
LSNS-6 sum score, *M* (SD)	17.5 (5.1)	17.7 (4.8)	18.1 (5.2)	17.3 (5.7)	13.3 (5.4)	67.9	**<0.001**
Anxiety							
GAD-7 sum score, *M* (SD)	3.5 (3.2)	3.3 (3.2)	3.8 (3.1)	3.8 (2.9)	5.2 (4.3)	29.0	**<0.001**

*Note: M*, SD, and % are weighted by age and sex according to census data and *n* is unweighted counts. Anxiety symptoms and social resources were measured with the GAD-7, and the short version of the LSNS-6. Bold *p*-values indicate significance. Education was assessed according to CASMIN-categories low, middle, and high. *N* = sample size; *M* = mean value; SD = standard deviation; *F* = *F* statistic, Chi^2^ = chi-square statistic. FTE group = full-time employment, PTE group = part-time employment, ALG I group = being unemployed receiving entitlement-based benefits, ALG II group = being unemployed receiving means-tested benefits.

Abbreviations: CASMIN, Comparative Analysis of Social Mobility in Industrial Nations; GAD-7, General Anxiety Disorder Scale-7; LSNS-6, Lubben Social Network Scale 6.

*⁣* ^*∗*^Comparisons with the employment status groups.

**Table 2:** Prevalence of anxiety—overall and by employment status group and sex.

Variable	Overall, *N* = 4885	CI (95%)	FTE group, *n* = 3664	95% CI	PTE group, *n* = 769	95% CI	ALG I group, *n* = 134	95% CI	ALG II group, *n* = 318	95% CI	Chi^2^*⁣*^*∗*^	*p*-Value
Anxiety (sum score GAD-7 ≥10), *n* (%)	292 (5.6)	5.0–6.3	183 (5.0)	4.3–5.7	53 (4.8)	3.5–6.5	5 (2.7)	0.8–7.0	51 (17.6)	13.3–22.4	75.3	**<0.001**
Sex												
Anxiety in males (sum score GAD-7 ≥10), *n* (%)	90 (3.6)	3.0–4.5	63 (2.9)	2.2–3.8	3 (4.0)	1.7–8.1	2 (1.9)	0.2–8.5	22 (13.6)	8.7–20.0	27.8*⁣*^*∗∗*^	**<0.001**
Anxiety in females (sum score GAD-7 ≥10), *n* (%)	202 (7.6)	6.5–8.7	120 (7.6)	6.3–9.0	50 (5.0)	3.5–7.0	3 (3.4)	0.7–10.6	29 (22.0)	15.3–29.9	32.8*⁣*^*∗∗*^	**<0.001**

*Note:* % are weighted by age and sex according to census data and *n* are unweighted counts. FTE group = full-time employment, PTE group = part-time employment, ALG I group = being unemployed receiving entitlement-based benefits, ALG II group = being unemployed receiving means-tested benefits. Bold *p*-values indicate significance. Chi^2^ = chi-square statistic. *N*/*n* = sample size.

Abbreviations: CI, confidence interval; GAD-7, General Anxiety Disorder Scale-7.

*⁣* ^*∗*^Comparisons with the employment status groups.

*⁣* ^*∗∗*^Fisher's exact test applied due to small cell sizes.

**Table 3:** Results of negative binomial regressions for the association between employment status groups, sociodemographic, social resources, and anxiety symptoms (*N* = 4885).

Variable	Model 1					Model 2					Model 3			
	IRR	95% CI	Wald/Chi^2^	*p*-Value	IRR		95% CI	Wald/Chi^2^	*p*-Value	IRR		95% CI	Wald/Chi^2^	*p*-Value
Employment status groups														
Employment status	—	—	44.99	**<0.001**	—		—	29.39	**<0.001**	—		—	17.25	**<0.001**
FTE group = Ref.	—	—	—	—	—		—	—	—	—		—	—	—
PTE group	1.14	1.04–1.25		**0.006**	1.04		0.94–1.15		0.435	1.03		0.94–1.12	—	0.495
ALG I group	1.13	0.96–1.33		0.153	1.08		0.90–1.29		0.399	1.07		0.91–1.25	—	0.403
ALG II group	1.55	1.36–1.78		**<0.001**	1.43		1.25–1.62		**<0.001**	1.29		1.14–1.45	—	**<0.001**
Sociodemographic variables														
Sex														
Male = Ref.	—	—	—	—	—		—	—	—	—		—	—	—
Female	—	—	—	—	1.37		1.26–1.48	—	**<0.001**	1.38		1.28–1.49	—	**<0.001**
Age					1.00		1.00–1.01	—	0.168	1.00		1.00–1.00	—	0.938
Education								16.49	**<0.001**				9.85	**0.007**
Low = Ref.	—	—	—	—	—		—	—	*—*	—		—	—	—
Middle	—	—	—	—	0.73		0.63–0.86	—	**<0.001**	0.78		0.67–0.91	—	**0.002**
High	—	—	—	—	0.73		0.62–0.85	—	**<0.001**	0.80		0.68–0.94	—	**0.006**
Marital status								12.14	**0.016**				6.25	0.181
Married living together = Ref.	—	—	—	—	—		—	—	—	—		—	—	—
Married living separately	—	—	—	—	1.19		1.03–1.39	—	**0.022**	1.14		0.98–1.32	—	0.094
Single	—	—	—	—	1.08		0.98–1.18	—	0.107	1.03		0.94–1.13	—	0.564
Divorced	—	—	—	—	1.13		1.03–1.23	—	**0.008**	1.08		0.99–1.17	—	0.076
Widowed	—	—	—	—	0.95		0.78–1.16	—	0.621	0.92		0.76–1.13	—	0.443
Social resources														
LSNS-6	—	—	—	*—*	—		—	—	*—*	0.97		0.97–0.98	—	**<0.001**
*R* ^2^	0.003				0.011					0.015				

*Note:* Results are weighted by age and sex according to census data. Anxiety symptoms and social resources were measured with the GAD-7 and the short version of the Lubben Social Network Scale 6 (LSNS-6). Bold *p*-values indicate significance. Education was assessed according to CASMIN-categories low, middle, and high. *N* = sample size, Chi^2^ = Chi^2^ statistic. *R*^2^ = proportion of variance in the dependent variable. FTE group = full-time employment, PTE group = part-time employment, ALG I group = being unemployed receiving entitlement-based benefits, ALG II group = being unemployed receiving means-tested benefits.

Abbreviations: CASMIN, Comparative Analysis of Social Mobility in Industrial Nations; CI, confidence interval; GAD-7, General Anxiety Disorder Scale-7; IRR, incidence rate ratio.

## Data Availability

The data that support the findings of this study are not publicly available due to privacy and ethical restrictions, but they are available from the corresponding author upon reasonable request.

## Supporting Information

Supporting Information The Supporting Information comprises three additional tables. Table S1 presents the findings of the logistic regressions examining sex differences in the prevalence of anxiety. Table S2 illustrates the results of the negative binomial regressions investigating the relationship between employment status groups, sociodemographic variables, social resources, and anxiety symptoms, separately by sex. Table S3 shows the results of the additional negative binomial regression, including depressive symptoms as an additional predictor variable.
