# Supplementary material for: The Relationship Between Anxiety and Employment Status in a German Working-Age Population: Findings on Sex-Specific Prevalence Rates and Associated Factors of Anxiety From the LIFE-Adult-Study
Source: Depress Anxiety. 2025 Jul 30;2025:1883623. doi: 10.1155/da/1883623 (PMC12328056; doi:10.1155/da/1883623)
Supplement: Supporting Information — The Supporting Information comprises three additional tables. Table S1 presents the findings of the logistic regressions examining sex differences in the prevalence of anxiety. Table S2 illustrates the results of the negative binomial regressions investigating the relationship between employment status groups, sociodemographic variables, social resources, and anxiety symptoms, separately by sex. Table S3 shows the results of the additional negative binomial regression, including depressive symptoms as an additional predictor variable. [file 1883623.f1.docx]

Supplementary material

Table S1. Results of logistic regressions for sex differences in prevalence of anxiety

|  | Overall N = 4,885 | | | FTE group n = 3,664 | | | PTE group n = 769 | | | ALG I group n = 134 | | | ALG II group n = 318 | | |
| --- | --- | --- | --- | --- | --- | --- | --- | --- | --- | --- | --- | --- | --- | --- | --- |
| Variable | OR | 95 % CI | p-value | OR | 95 % CI | p-value | OR | 95 % CI | *p-value* | OR | 95 % CI | *p-value* | OR | 95 % CI | *p-value* |
| Sex  Male = Ref.  Female | 2.14 | 1.63-2.80 | **<.001** | 2.69 | 1.94-3.73 | **< .001** | 1.30 | 0.53-3.23 | .565 | 1.31 | 0.13-3.23 | .817 | 1.73 | 0.91-3.29 | .094 |

*Notes. Results are weighted by age and sex according to census data. Anxiety symptoms were measured with the General Anxiety Disorder Scale-7 (GAD-7, cut-off* *≥ 10). Bold p-values indicate significance. N = sample size, OR = odds ratio, CI = confidence interval. Ref. = reference group.* *FTE group = full-time employment, PTE group = part-time employment, ALG I group = being unemployed receiving entitlement-based benefits, ALG II group = being unemployed receiving means-tested benefits.*

Table S2. Results of negative binomial regressions for the association between employment status groups, sociodemographic variables, social resources and anxiety symptoms by sex

|  | Female n = 2627 | | | | Male n = 2258 | | | |
| --- | --- | --- | --- | --- | --- | --- | --- | --- |
| Variable | IRR | 95 % CI | Wald/Chi^2^ | *p-value* | IRR | 95 % CI | Wald/Chi^2^ | *p-value* |
| Employment status groups |  |  |  |  |  |  |  |  |
| Employment status  FTE group = Ref.  PTE group  ALG I group  ALG II group | 0.96  1.00  1.23 | 0.87-1.07  0.85-1.19  1.04-1.45 | 7.39 | .060  .477  .961  **.015** | 1.31  1.15  1.36 | 1.06-1.62  0.87-1.52  1.14-1.62 | 17.23 | **<.001**  **.013**  .320  **.001** |
| Sociodemographic variables |  |  |  |  |  |  |  |  |
| Age | 1.00 | 0.99-1.00 |  | .611 | 1.00 | 1.00-1.01 |  | .569 |
| Education  Low = Ref.  Middle  High | 0.81  0.82 | 0.66-0.98  0.66-1.00 | 4.52 | .104  **.034**  .055 | 0.78  0.79 | 0.63-0.96  0.64-0.98 | 5.65 | .059  **.018**  **.036** |
| Marital status  Married living together = Ref.  Married living separately  Single  Divorced  Widowed | 1.03  1.03  1.08  0.89 | 0.85-1.24  0.91-1.17  0.98-1.20  0.71-1.11 | 3.96 | .412  .779  .632  .121  .295 | 1.28  1.00  1.06  1.06 | 1.01-1.61  0.87-1.15  0.93-1.21  0.68-1.66 | 4.80 | .308  .**041**  .981  .398  .792 |
| Social resources |  |  |  |  |  |  |  |  |
| LSNS-6 | 0.97 | 0.96-0.98 |  | **<.001** | 0.98 | 0.96-0.99 |  | **<.001** |
| R² | .011 | | | | .010 | | | |

*Notes. Results are weighted by age and sex according to census data. Anxiety symptoms and social resources were measured with the General Anxiety Disorder Scale-7 (GAD-7) and the short version of the Lubben Social Network Scale 6 (LSNS-6). Bold p-values indicate significance. Education was assessed according to CASMIN (Comparative Analysis of Social Mobility in Industrial Nations) - categories low, middle, and high. n = sample size, IRR = incidence rate ratio, CI = confidence interval, Chi^2^ = Chi^2^ statistic. R^2^ = proportion of variance in the dependent variable. Ref. = reference group. FTE group = full-time employment, PTE group = part-time employment, ALG I group = being unemployed receiving entitlement-based benefits, ALG II group = being unemployed receiving means-tested benefits.*

Table S3. Results of negative binomial regressions for the association between employment status groups, sociodemographic, social resources, depression and anxiety symptoms (N = 4,885)

|  | model 1 | | | | | model 2 | | | | | model 3 | | | |
| --- | --- | --- | --- | --- | --- | --- | --- | --- | --- | --- | --- | --- | --- | --- |
| Variable | IRR | 95 % CI | Wald/Chi^2^ | *p-value* | IRR | | 95 % CI | *Wald/Chi^2^* | *p-value* | IRR | | 95 % CI | Wald/Chi^2^ | *p-value* |
| Employment status groups |  |  |  |  |  | |  |  |  |  | |  |  |  |
| Employment status  FTE group = Ref.  PTE group  ALG I group  ALG II group | 1.14  1.13  1.55 | 1.04-1.25  0.96-1.33  1.36-1.78 | 44.99 | **< .001**  **.006**  .153  **< .001** | 1.04  1.08  1.43 | | 0.94-1.15  0.90-1.29  1.25-1.62 | 29.39 | **< .001**  .435  .399  **< .001** | 1.03  0.87  0.96 | | 0.94-1.12  0.75-1.01  0.87-1.04 | 5.07 | .167  .548  .063  .309 |
| Sociodemographic variables |  |  |  |  |  | |  |  |  |  | |  |  |  |
| Sex  Male = Ref.  Female |  |  |  |  | 1.37 | | 1.26-1.48 |  | **< .001** | 1.18 | | 1.11-1.25 |  | **<.001** |
| Age |  |  |  |  | 1.00 | | 1.00-1.01 |  | .168 | 1.00 | | 1.00-1.00 |  | .632 |
| Education  Low = Ref.  Middle  High |  |  |  |  | 0.73  0.73 | | 0.63-0.86  0.62-0.85 | 16.49 | **< .001**  **< .001**  **< .001** | 0.88  0.98 | | 0.76-1.01  0.85-1.14 | 14.75 | **<.001**  .077  .830 |
| Marital status  Married living together = Ref.  Married living separately  Single  Divorced  Widowed |  |  |  |  | 1.19  1.08  1.13  0.95 | | 1.031.39  0.98-1.18  1.03-1.23  0.78-1.16 | 12.14 | **.016**  **.022**  .107  **.008**  .621 | 0.95  0.93  0.99  0.80 | | 0.84-1.08  0.86-1.00  0.93-1.06  0.70-0.91 | 14.04 | **.007**  .455  .056  .818  **.001** |
| Depression |  |  |  |  |  | |  |  |  |  | |  |  |  |
| CES-D |  |  |  |  |  | |  |  |  | 1.07 | | 1.06-1.07 |  | **<.001** |
| Social resources |  |  |  |  |  | |  |  |  |  | |  |  |  |
| LSNS-6 |  |  |  |  |  | |  |  |  | 1.00 | | 0.99-1.00 |  | .321 |
| R² | .003 | | | | .011 | | | | | .090 | | | | |

Notes. Results are weighted by age and sex according to census data. Depressive symptoms, anxiety symptoms and social resources were measured with the Center for Epidemiologic Studies Depression Scale (CES-D), the General Anxiety Disorder Scale-7 (GAD-7), and the short version of the Lubben Social Network Scale 6 (LSNS-6). Bold p-values indicate significance. Education was assessed according to CASMIN (Comparative Analysis of Social Mobility in Industrial Nations) - categories low, middle and high. N = sample size, IRR = incidence rate ratio, CI = confidence interval, Chi^2^ = Chi^2^ statistic. R^2^ = proportion of variance in the dependent variable. FTE group = full-time employment, PTE group = part-time employment, ALG I group = being unemployed receiving entitlement-based benefits, ALG II group = being unemployed receiving means-tested benefits.

Depressive Symptoms

Depressive symptoms were assessed using the German version of the Center for Epidemiological Studies Depression Scale (CES-D), which consists of 20 items assessing symptoms such as hopelessness, depressive mood, and uncertainty experienced in the last week (Radloff, 1977; Hautzinger et al., 2012). The scale is a widely used measuring instrument. A 4-point Likert scale was used to answer the items (0 = 'rarely or none of the time', 1 = 'some or a little of the time', 2 = 'occasionally or a moderate amount of time', and 3 = 'most or almost all of the time'). The total score ranges from 0 to 60, with higher values indicating more pronounced symptoms. The cut-off value for depression is ≥ 23.
